# Pore network modeling of water and gas transport characteristics in synthetic porous media

**DOI:** 10.1371/journal.pone.0341834

**Published:** 2026-01-30

**Authors:** Li Dong, Changkun Ma, Wanghai Tao, Quanjiu Wang

**Affiliations:** State Key Laboratory of Water Engineering Ecology and Environment in Arid Area, Xi’an University of Technology, Xi’an, P. R. China; Jeddah University: University of Jeddah, SAUDI ARABIA

## Abstract

The flow characteristics of water and gas are closely linked to pore structure of porous media, which is of critical importance across various scientific and industrial fields. In this study, synthetic porous media with varying grain sizes and porosity were generated, and their corresponding pore structures were characterized using pore network modeling. Furthermore, the intrinsic permeability, water retention curve, water-gas relative permeability and relative gas diffusivity of the synthetic porous media were simulated via pore network modeling. The results demonstrate that the pore networks extracted from images can effectively distinguish pore structural characteristics. Specifically, the mean pore diameter, throat diameter, and throat length were larger in coarse-grained media compared to fine-grained media of the same porosity. In contrast, fine-grained media exhibited higher values for pore number, throat number, and coordination number. Additionally, the distributions of pore diameter, throat diameter, throat length and coordination were found to follow a lognormal distribution. Porous media with coarse grains and larger porosity exhibit greater intrinsic permeability and relative gas diffusivity compared to media composed of finer grains or lower porosity. The water-retention curves were fitting by van Genuchten model, revealing an exponential relationship between parameter α and throat diameter (or pore diameter). But the parameter *n* did not show a clear trend across various synthetic porous media, which is attributed to the relatively narrow range of pore size distribution. Similarly, for water-gas relative permeability, the critical water saturation did not vary significantly across different porous media. A strong correlation was observed among the pore structural parameters, irrespective of grain shape and size. Both intrinsic permeability and relative gas diffusivity exhibited a power-law relation with the porosity as well as with pore or throat radius. Moreover, the relationship between intrinsic permeability and relative gas diffusivity can be expressed as *k* = 166.51(*D*_*p*_/*D*_*0*_)^0.98^, which provides a direct means of estimating relative gas diffusion from intrinsic permeability directly.

## 1. Introduction

Porous media, a type of material composed of pore space and solid skeletons, are widely present across a range of scales in various scientific and industrial fields, including groundwater contamination remediation, hydrocarbon resources reservoirs, subsurface systems for CO_2_ sequestration and filtration processes [1]. In particular, pore structure of porous media governs the volumes of water and gas present, energy status, diffusivity, and the saturated/unsaturated transport of gas, water, and solutes [2,3]. For instance, the distribution of efflorescence and subflorescence in the porous materials is influenced by the internal pore structure [4]. Pore characteristics also shape the transport of agrochemicals, aeration and biochemical in agricultural field [5]. Similarly, the transport of soil gases and atmospheric exchange, which affects greenhouse gas emissions, is tightly linked to the pore structure of diverse porous media [6]. Therefore, a thorough understanding and quantification of pore structures properties, such as pore size, pore connectivity, pore distribution, and their impact on flow and transport processes are essential.

Accurate measurement and characterization of pore structure are essential prerequisite for investigating flow and transport properties of porous media. Traditionally, pore structure characteristics such as pore size and distribution have been inferred indirectly through methods including the water retention curve [7], gas adsorption [8], and mercury intrusion porosimeter [9]. However, these conventional techniques are destructive, time- and labor-intensive, and cannot capture the spatial, morphological, or topological features of pore structure. In recent years, many non-destructive methods have been emerged and applied, such as X-ray computed tomography, scanning electron microscopy, and magnetic resonance imaging, for revealing the three-dimensional pore structure of porous media [10,11]. Through a series of digital image processing steps, such as noise reduction via filtering, phase contrast enhancement, and segmentation of grayscale data into two or more phases, the volumes of gas, liquid, and solid can be distinguished. The extracted pore structure has been further quantified using pore network models, which simplifies complex pore systems into a disarrayed or arrayed three-dimensional network of pore bodies and pore throats [10]. Key quantitative parameters include pore body radius, pore throat radius, and throat length, which characterize pore size distribution and spatial arrangement, as well as coordination number, which indicates connectivity by representing the number of throats connected to a given pore. Pore shapes analysis has also been conducted via digital images analysis [12]. Nevertheless, the above methods for obtaining pore structure images of porous media remains costly, computationally demanding and not widely accessible [13]. In addition, the corresponding images cannot contain all pore spaces information due to the trade-off between resolution and images size [14,15]. As a result, how to generate images of pore structure directly and accurately based on numerical method is still a challenging task.

The flow and transport properties of porous media across different scales are fundamentally governed by pore structure, and numerous numerical simulations have been conducted to explore this relationship. Three-dimensional representations of pore space have been reconstructed with varied pore-size distributions and connectivity to predict essential hydraulic characteristics. Compared to other pore-scale models, such as Lattice-Boltzmann and particle methods, pore network model is more effective for simulating complex transport processes in porous media, requiring less computational time and resources [1]. Additionally, pore network model can provide pore-scale distribution of variables such as velocity, pressure, temperature, and concentration, which are challenging to measure experimentally [16]. Previous studies have demonstrated that hydraulic conductivity increased significantly with pore diameter, particularly when pore diameter exceeds 50 μm [17]. Base on experimental investigations, Luo et al [18] found that hydraulic conductivity is larger controlled by the number of independent and continuous macropore paths. Saturated hydraulic conductivity has also been effectively estimated using critical radius of pore or throat, and connectivity [19]. But the application of these findings across diverse media remains limited. Furthermore, two-phase flow (e.g., water and gas) is universal in natural porous system, with water generally occupying smaller pores and gas migrating through larger pores. Consequently, the spatial arrangement of pore size significantly influences water-gas exchange efficiency, where the relative permeability to gas is governed connectivity of pore size distribution [20]. Recently, Wei et al [21] observed that pore size distribution is closely related to gas compression induced by critical infiltration rate, through the underlying mechanisms are still not fully understood.

The present study serves as a preliminary step in the generation and application of pore networks derived from synthetic porous media. Its objectives are to generate diverse pore network from artificial images and to quantify the influence of pore structure on transport characteristics across different porous media, including the identification of general relationships between permeability and pore-structure parameters. To achieve these purposes, porous media with varied structures were constructed using artificially generated images representing distinct morphologies. The pore structure was quantitatively characterized, and the transport properties of different porous media were simulated through pore network modeling.

## 2. Materials and methods

### 2.1. Pore network construction

**Fig 1:**
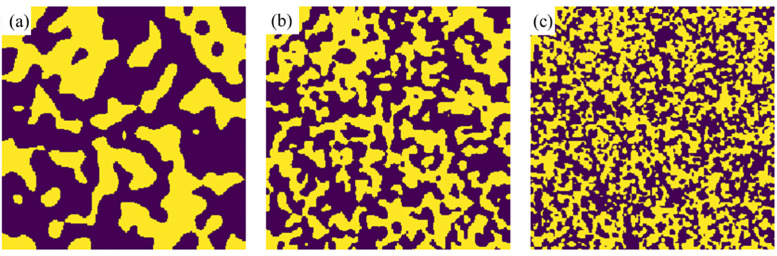
Binary images of synthetic porous media with blobs sizes of (a) 1, (b) 2 and (c) 4, where yellow parts represent void and the black are solid (The above figures were generated through PoreSpy v2.3.0, which can be installed from https://github.com/PMEAL/porespy).

Pore networks typically characterize pore structure through parameters such as pore body distribution, pore throat distribution, throat length, and coordination number, which can be extracted from binary images using the subnetwork of the over segmented watershed (SNOW) algorithm [22]. In this study, the “blobs” model within PoreSpy package was employed to generate artificial images through creating random noise, applying Gaussian blur, renormalizing the result to a uniform distribution, and thresholding to produce a binary image. The porous media structure was varied by adjusting porosity and blob size; larger blob sizes correspond to a greater number of small, randomly distributed blobs in the image, which simulates smaller particle size in the porous media. Using the method described above, a series of images with a voxel size of 20 μm were generated. For instance, Fig 1 shows representative slices of 3D porous media with a porosity of 0.45 and blob sizes of 1, 2, and 4 respectively. Moreover, tortuosity, defined as the ratio of the actual flow path through the pore space to the straight-line distance across the medium, is a key parameter influencing the flow and transport characteristics of porous media. Tortuosity was calculated using a finite difference method implemented in the PoreSpy package. Specific methodological details can be referred to Gostick et al [23]. Pore identification and network generation were performed using the SNOW algorithm, and transport characteristics within the extracted networks were simulated with OpenPNM [24]. The influences of pore structures on flow properties were subsequently analyzed through these pore networks.

**Fig 2:**
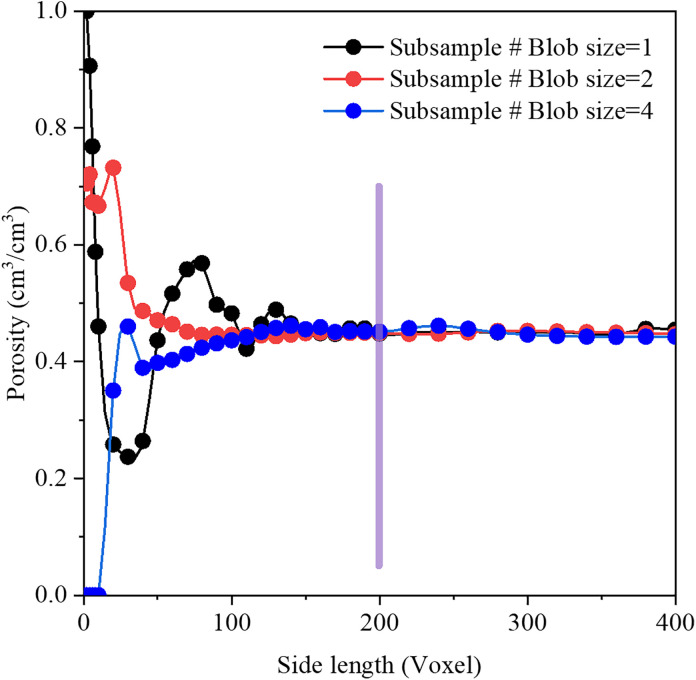
Determination of an appropriate representative elementary volume from porosity.

**Fig 3:**
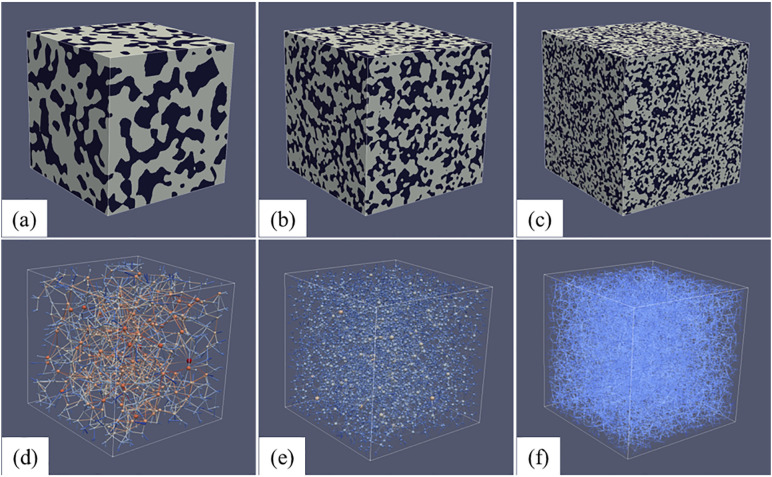
Imaging data for synthetic porous media with size of 4 × 4 × 4 mm^3^, and equivalent unstructured pore network from SNOW network extraction code: (a) and (d) with blobs size of 1, (b) and (e) with blobs size of 2, and (c) and (f) with blobs size of 4, respectively (The above figures rendered through software of ParaView v5.13, which can be downloaded from https://www.paraview.org).

To examine the effects of structural characteristics, nine types of synthetic images were generated with varying blob sizes (1, 2, and 4) and porosities (0.35, 0.45, and 0.55), as summarized in Table 1. Each configuration was replicated three times to minimize error. Porosity was also used as an indicator to determine the representative elementary volume (REV). For porous media with a fixed porosity of 0.45 but different blob sizes, porosity was measured from three independent subsample with varying side lengths. As shown in Fig 2, porosity began to stabilize and exhibited REV characteristics once the cube side length exceeded approximately 150 voxels. Therefore, for simplicity and consistency, all REVs were selected as cubes of 200 × 200 × 200 voxel^3^, corresponding to a physical dimension of 4 × 4 × 4 mm^3^ in this study. Pore networks of each cube were extracted from SNOW network extraction code and then can be rendered through ParaView software, as illustrated in Fig 3.

**Table 1:** Statistics of the parameters to quantify the pore structure of synthetic porous media.

Blobs size	Porosity (%)	Tortuosity (-)			Pore number (-)	Throat number (-)	Average pore diameter (μm)	Average throat diameter (μm)	Average throat length (μm)	Coordination number (-)
		*X* and *Y*- horizontal								*Z*-vertical
1	34.81 ± 0.30	3.77 ± 0.05	3.66 ± 0.08	3.61 ± 0.09	1206 ± 42	1942 ± 98	277.75 ± 2.67	112.28 ± 0.94	57.83 ± 4.79	3.22 ± 0.55
	44.59 ± 0.37	3.20 ± 0.04	3.13 ± 0.07	3.15 ± 0.05	1269 ± 27	2331 ± 88	294.06 ± 0.25	121.97 ± 0.31	56.63 ± 1.76	3.67 ± 0.06
	55.20 ± 0.38	2.77 ± 0.16	2.72 ± 0.11	2.69 ± 0.20	1259 ± 51	2662 ± 137	313.26 ± 3.28	132.22 ± 1.37	59.17 ± 2.52	4.23 ± 0.06
2	34.18 ± 0.13	3.01 ± 0.08	2.98 ± 0.08	2.97 ± 0.08	5355 ± 83	9584 ± 182	172.70 ± 0.64	69.35 ± 0.15	41.15 ± 0.33	3.58 ± 0.01
	44.36 ± 0.24	2.60 ± 0.02	2.59 ± 0.05	2.59 ± 0.04	5483 ± 28	11839 ± 149	185.36 ± 0.68	75.72 ± 0.29	41.33 ± 0.70	4.32 ± 0.04
	54.46 ± 0.25	2.24 ± 0.02	2.24 ± 0.10	2.25 ± 0.12	5354 ± 42	13559 ± 61	198.92 ± 1.38	82.00 ± 0.49	40.50 ± 0.52	5.05 ± 0.04
4	35.14 ± 0.05	2.45 ± 0.03	2.46 ± 0.02	2.44 ± 0.04	15987 ± 110	35294 ± 162	121.74 ± 0.30	48.83 ± 0.08	40.21 ± 0.24	4.42 ± 0.02
	44.68 ± 0.10	2.10 ± 0.03	2.10 ± 0.02	2.11 ± 0.02	15996 ± 82	43231 ± 64	129.90 ± 0.39	52.99 ± 0.11	38.94 ± 0.42	5.41 ± 0.03
	54.17 ± 0.09	1.80 ± 0.04	1.81 ± 0.01	1.81 ± 0.03	15681 ± 95	49215 ± 178	138.07 ± 0.44	57.22 ± 0.12	37.70 ± 0.19	6.28 ± 0.04

### 2.2. Simulation of flow and transport property

Relative permeability is important for estimating the flow of reservoir fluids in porous media, which defines as dimensionless functions of saturation. The following assumptions were made in flow simulations with pore-network: the throats were assumed as cylindrical pips, perfect fluid mixing was assumed to occur within pores and flow through throats was considered laminar flow. Consequently, fluids flow in throats can be described by the Hagen-Poiseuille equation,


$$q_{ij}=\frac{\pi {R_{ij}}^{4}\left(P_{i}-P_{j}\right)}{8\mu L_{ij}}$$ (1)

where *q*_*ij*_, *R*_*ij*_ and *L*_*ij*_ are the flow rate, the radius and length of the throat, *P*_*i*_ and *P*_*j*_ are the pressures in the pores *i* and *j*, and *μ* is the ﬂuid viscosity, which is 1.0 × 10^−3^ Pa·s for water.

The sum of flow in and out is zero for each pore,


$$\sum_{j=p_{1}}^{p_{n}}q_{ij}=0$$ (2)

where *p*_*1*_,…, *p*_*n*_ are pores. Solving Eq. (2) can obtain each pore pressure, and then calculated flow rate through each throat. The macroscopic flow rate can also be calculated through summing flux over one boundary pores. For single-phase flow, the intrinsic permeability can be derived from these results,


$$k=\frac{\mu QL}{A(P_{in}-P_{out})}$$ (3)

where *Q* is the inlet flow rate with the condition of single phase, *A* is the inlet area, *L* is the distance between inlet and outlet, *P*_*in*_ and *P*_*out*_ are the pressures in boundary of inlet and outlet, which are set as 100 kPa and 0 kPa respectively.

When two phases, such as gas and water, coexist in the pore network, the invasion percolation algorithm was employed to determine phase invasion sequence. As gas progressively invades the network, the phase occupancy at each saturation was update to compute the flow rates of both gas and water phases. The capillary pressure threshold for invasion in each throat is given by Laplace’s law, i.e., $P_{c}=\frac{\text{2}\gamma \text{cos}\theta }{r}$, where *γ* is surface tension, *θ* is equilibrium contact angle and *r* is throat radius. For the application of the invasion percolation algorithm, the contact angle was assumed constant throughout the media and significantly less than 90^◦^ [25]. In this study, pore body and throat were simplified sphere and cylinder; therefore, the contact angle was set to zero. Relative permeability is generally defined as the ratio of effective permeability to absolute permeability. Under identical boundary conditions, fluid viscosities and geometrical parameters, it can be expressed as,


$$k{\mathrm{\backslash nolimits}}_{r}=\frac{k{\mathrm{\backslash nolimits}}_{eff}}{k}=\frac{Q{\mathrm{\backslash nolimits}}_{eff}}{Q}$$ (4)

where *Q*_*eff*_ is inlet flow rate of the phase with condition of multiphase.

The water retention curves of porous media were simulated through pore network model. The invasion percolation algorithm was adapted to simulate the gradual displacement of water by gas in the initially water-saturated network, neglecting viscous effects. That is, gas generally invades the largest pore at boundary firstly, and the smaller pores are invaded with the increasing of pressure which responds to one saturation. Thus, the water retention curves could be obtained through pressure and saturation until equilibrium. The main steps include, 1) Identifying the largest throat in water and gas interface, and obtaining gas entry pressure through Young-Laplace Equation 2) Comparing the difference pressure between capillary pressure and gas-entry pressure. The gas will invade the throat saturated with water if capillary pressure exceeds the gas-entry pressure. 3) Updating the distribution of water and gas, and repeating the calculation processed in condition of new pressures. All simulations were carried out with the OpenPNM package [24]. The resulting water retention dates were subsequently fitted using the van Genuchten model [26].

Porous media gas diffusivity is an important parameter indicative of the diffusion mobility. Gas diffusion is commonly characterized by relative gas diffusivity, *D*_*p*_/*D*_0_, where *D*_*p*_ (m^3^ porous media air m^-1^ porous media s^-1^) and *D*_0_ (m^2^ air s^-1^) are given gas diffusion coefficients in porous media and in free air, respectively. Diffusion of gas in throats can be described by Fick’s first law,


$$J{\mathrm{\backslash nolimits}}_{ij}=-\frac{D{\mathrm{\backslash nolimits}}_{v}A{\mathrm{\backslash nolimits}}_{ij}\Delta C{\mathrm{\backslash nolimits}}_{ij}}{L{\mathrm{\backslash nolimits}}_{ij}}=-\frac{D{\mathrm{\backslash nolimits}}_{v}A{\mathrm{\backslash nolimits}}_{ij}}{L{\mathrm{\backslash nolimits}}_{ij}}\left(C{\mathrm{\backslash nolimits}}_{i}-C{\mathrm{\backslash nolimits}}_{j}\right)$$ (5)

where *J*_*ij*_ is the diffusive mass flux, *C*_*ij*_ is the throat concentration connected with pore *C*_*i*_ and pore *C*_*j*_, respectively, *A*_*ij*_ and *L*_*ij*_ are the area and length of the throat. *D*_*v*_ is the diffusion coefficient of vapor in the air and this is the diffusion coefficient in an open space, not the effective diffusivity. In the vapor diffusion simulation, the system was assumed to be isothermal and isobaric, and the gravity influence was neglected. The vapor diffusion in each pore, assuming steady-state, is expressed as,


$$\sum_{j=p_{1}}^{p_{n}}J_{ij}=0$$ (6)

Each pore concentration can be obtained by solving Eq. (6), and diffusive mass flux in each throat also can be calculated. The gas diffusion coefficients in porous media (*D*_*p*_) of the network can be determined by solving Fick’s law as,


$$D{\mathrm{\backslash nolimits}}_{p}=\frac{JL}{A\Delta C}$$ (7)

where *J* is the inlet diffusive mass flux of vapor, and Δ*C* is the concentration difference between inlet and outlet, which is set to one. To obtain the relative gas diffusivity, the *D*_*p*_ was then scaled with the gas diffusion coefficient in free air (*D*_0_ = 2.05 × 10^−5^ m^2^ s^-1^).

## 3. Results and discussion

### 3.1. Pore structure of synthetic porous media

Synthetic binary images of porous media with various blobs sizes were generated through PoreSpy as shown in Fig 1, where yellow parts represent void space and black space are solid grains. Porus media with blob sizes of 1 and 4 represent coarse- and fine-grained structures, respectively, while a blob size of 2 corresponds to an intermediate grain size. Table 1 presents the macro- and micro-structural parameters of pore structure. The macro porosity values ranged closely around the predefined targets of 35%, 45% and 55%, confirming that the generated media accurately match the intended porosity levels. Tortuosity showed negligible variation between horizontal (*X*- and *Y*- directions) and vertical (*Z*- direction in Cartesian coordinates) directions across all synthetic media (Table 1). To assess the directional dependence of tortuosity, measurements were taken on 27 independent samples along three orthogonal directions (horizontal *X* and *Y*, vertical *Z*). After controlling for inter-sample variability using a repeated-measures analysis of variance, a significant effect of direction on tortuosity was found, that is *F*(2, 52)=7.24, *p* = 0.002. Specifically, tortuosity in the horizontal *X*-direction (2.730 ± 0.121) was significantly higher than in the horizontal *Y*-direction (2.724 ± 0.119, *p* = 0.028) and the vertical Z-direction (2.724 ± 0.120, *p* = 0.028), while no significant difference was observed between the latter two directions (*p* = 0.995). Meanwhile, error quantification revealed that neglecting this anisotropy and using an isotropic average (*τ* = 2.726) introduces a maximum relative error of only 0.15%. Given that the precision requirements of the current model (typical experimental error over 5%) far exceed this value, and considering the clear anisotropic pattern (only one horizontal axis is slightly elevated), adopting the isotropic assumption as a simplification is both reasonable and acceptable. For each grain size, the lowest tortuosity occurred in samples with the highest porosity. Notably, coarse-grained media exhibited higher tortuosity than fine-grained media across all porosity levels.

Pore networks were extracted through the SNOW algorithm, with sphere representing pore bodies and cylinder representing throats. Fig 3 illustrates representative pore networks for different blob sizes; visually highlighting structural variations derived from these simplified geometries. Meanwhile, a set of parameters, including number of pore and throat, coordination number, diameter of pore and throat, and average throat length, were computed to statistically compare the networks (Table 1). Fine-grained media consistently showed the highest pore and throat counts, whereas the coarse ones yielded the lowest values at equivalent porosities. There was no significant difference in pore number for synthetic porous media with various porosity, but throat number increased with the increase of porosity for a given grain size.

**Fig 4:**
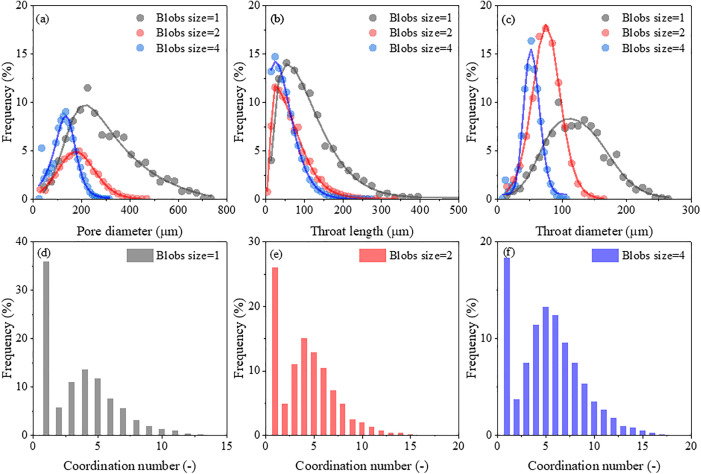
Distribution of pore-network parameters for synthetic porous media with porosity of 0.45: (a-c) distributions of pore diameter, throat length and throat diameter, and (d-f) coordination number distribution with different blobs sizes.

As expected, larger pores were observed in the synthetic porous media with coarser grains and higher porosity (Table 1), which aligns with earlier experimental and modeling results [27,28]. For coarse-grained media, the mean pore diameters were 277.75, 294.06 and 313.26 μm at porosities of 0.35, 0.45 and 0.55, respectively. In contrast, fine-grained media showed corresponding mean pore diameters of 121.74, 129.90 and 138.07 μm. Medium-grained structures exhibited intermediate values of 172.70, 185.36 and 198.92 μm for the same porosity levels. In addition, the mean throat diameter was approximately 0.40 times the mean pore diameter across all synthetic porous media, consistent with the findings reported by Ren and Santamarina [29]. Average throat length was primarily influenced by grain size: a decrease in grain size led to a marked reduction in throat length at a given porosity. For instance, at a porosity of 0.35, the mean throat lengths were 56.63, 41.33, 38.94 μm for blobs sizes of 1, 2 and 4, respectively. Compared with artificial or natural porous media, the pore diameter of fine-grained samples are similar to those reported for loam soil (113.36 μm) by Dong et al [30], while coarse-grained samples resemble the pore size of 0.6–1.4 mm glass beads (336 μm) present in Xiong et al [28]. The distributions of pore and throat diameter, as well as throat length followed the lognormal distribution across various synthetic media, although frequency profiles varied with grain sizes (Figs 4a-c). This lognormal behavior is consistent with prior studies on diverse porous systems, including soil subjected to freeze-thaw cycles or organic amendment, and fibrous material [30–32].

The coordination number, a fundamental microscale descriptor of pore connectivity, generally represents the feature of network topology. Table 1 presents the mean coordination number in this study ranged from 3 to 7. For a given grain size (blob size), the mean coordination number increased with porosity. At constant porosity, medium-grained media exhibited intermediate coordination numbers, while coarse-grained media consistently showed lower mean coordination numbers than fine-grained media. The distribution of coordination numbers also followed a lognormal distribution, with frequency profiles varying across different grain sizes (Figs 4d-f). These findings indicate that synthetic porous media generated in this study effectively capture pore-structure characteristics representative of artificial or natural porous media.

### 3.2. Water flow characteristics of different porous media

#### 3.2.1. Intrinsic permeability.

Intrinsic permeability, which depends solely on material structure and not of the fluid properties, was estimated for the various synthetic porous media based on the extracted pore networks. The values of intrinsic permeability ranged from 1.20 μm^2^ to 5.56 μm^2^ in both vertical and horizontal directions as shown in Table 2. Intrinsic permeability increased notably with porosity for a given grain size. Across various grain sizes, coarse-grained media exhibited the highest intrinsic permeability, followed by medium-grained media, while fine-grained media consistently showed the lowest values in each direction. In addition, no distinct anisotropy was observed between horizontal and vertical directions. Therefore, the vertical directions were selected as representative for subsequent analysis.

**Table 2:** Transport characteristics of porous media with different porosity in vertical (Z) and horizontal (X and Y) directions.

Blobs size	Porosity (%)	Intrinsic permeability (μm^2^)			Relative gas diffusivity ×10^−2^ (-)		
		*Z*	*X*	*Y*	*Z*	*X*	*Y*
1	34.81 ± 0.30	2.63 ± 0.03	2.79 ± 0.02	2.77 ± 0.05	1.57 ± 0.02	1.57 ± 0.01	1.55 ± 0.03
	44.59 ± 0.37	4.29 ± 0.02	4.21 ± 0.02	4.21 ± 0.03	2.37 ± 0.03	2.37 ± 0.01	2.37 ± 0.01
	55.20 ± 0.38	5.46 ± 0.19	5.56 ± 0.02	5.59 ± 0.06	3.11 ± 0.05	3.12 ± 0.01	3.14 ± 0.03
2	34.18 ± 0.13	1.73 ± 0.02	1.66 ± 0.05	1.66 ± 0.03	0.94 ± 0.02	0.93 ± 0.03	0.93 ± 0.02
	44.36 ± 0.24	2.62 ± 0.03	2.74 ± 0.05	2.73 ± 0.03	1.52 ± 0.06	1.54 ± 0.03	1.53 ± 0.02
	54.46 ± 0.25	3.99 ± 0.17	3.82 ± 0.13	3.85 ± 0.17	2.11 ± 0.02	2.14 ± 0.07	2.16 ± 0.10
4	35.14 ± 0.05	1.30 ± 0.07	1.20 ± 0.10	1.22 ± 0.04	0.65 ± 0.01	0.67 ± 0.05	0.69 ± 0.02
	44.68 ± 0.10	1.81 ± 0.01	1.92 ± 0.06	1.88 ± 0.04	1.03 ± 0.02	1.08 ± 0.03	1.06 ± 0.02
	54.17 ± 0.09	2.75 ± 0.05	2.64 ± 0.16	2.70 ± 0.15	1.39 ± 0.05	1.48 ± 0.09	1.52 ± 0.09

**Fig 5:**
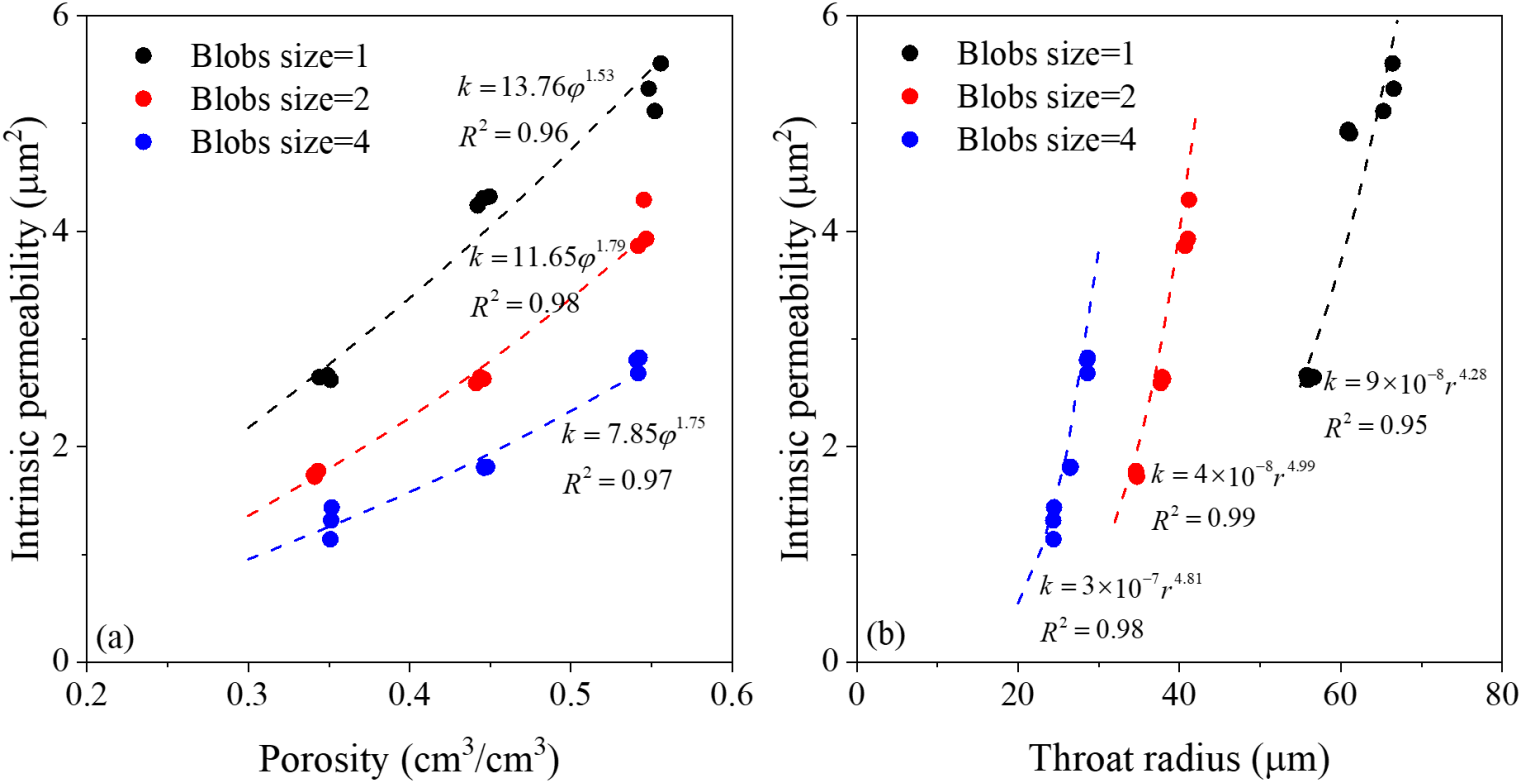
The relationship of porosity, throat radius and intrinsic permeability in different porous media (a) the intrinsic permeability vs. the porosity, and (b) the intrinsic permeability vs. throat radius.

The relationship between intrinsic permeability and porosity is present in Fig 5a. A power-law function accurately describes how intrinsic permeability varies with porosity across synthetic media of different morphologies. The fitted equations exhibit distinct coefficients for different grain sizes, indicating that grain size, distribution, and shape strongly influence intrinsic permeability. Previous studies also demonstrated that porosity, grain size and shape have influence on intrinsic permeability, where porosity is the most sensitive factor, and the next is grain size [33]. Fig 5a further shows that intrinsic permeability is more sensitive to porosity changes in coarse-grained media than in fine-grained media. Enhanced hydraulic conductivity in coarse-grained structures can be partly attributed to the presence of larger, randomly distributed pores, as shown in Fig 4a. However, the power-law equations used here express intrinsic permeability solely as a function of porosity, with other characteristics of pore structure (i.e., pore size, connectivity and distribution) are lumped in the fitted coefficients. Further, the micro-scale pore structure was considered to investigate the intrinsic permeability of porous media. As shown in Fig 5b, the relationship between intrinsic permeability and throat radius also can be expressed in power-law, consistent with findings by Gharedaghloo et al [34]. The coefficients of the power-law equation again differ with grain sizes, confirming that pore size, especially that of larger pores, is a crucial factor governing intrinsic permeability.

**Fig 6:**
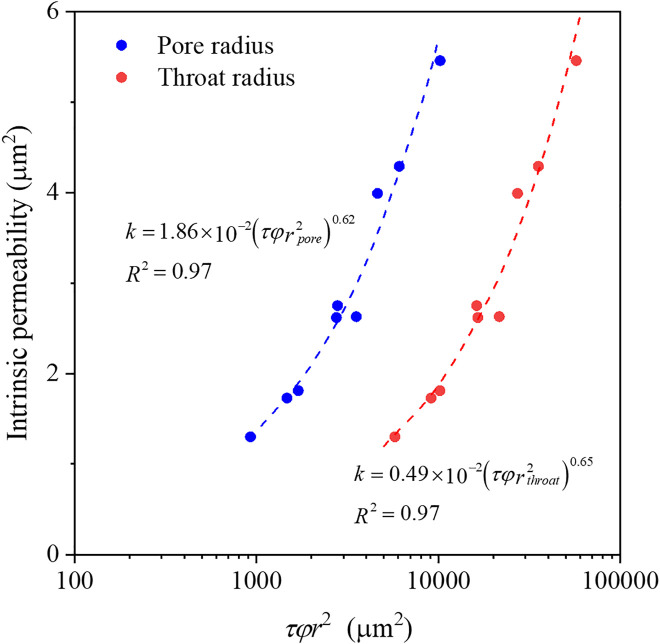
The intrinsic permeability as a function of *τφr*^2^ for porous media.


Furthermore, by considering both micro- and macro- structural parameters, the relationship between intrinsic permeability and pore structure can be expressed as,


$$k=c\varphi r{\mathrm{\backslash nolimits}}^{2}$$ (8)

where *c* is geometric factor related to pore shape and tortuosity, *φ* is porosity, and *r* is pore size. Since porous media contain pores with various sizes, an appropriate representative radius must be chosen for Equation 8. Common choices include the hydraulic radius (pore volume divided by wetted surface area), the pore radius corresponding to the gas‑entry pressure on the capillary pressure curve, or a radius derived from fractal‑percolation theory. In this study, the average pore (or throat) radius and tortuosity was used for pore size and geometric factor respectively, to calculate intrinsic permeability. Fig 6 shown the calculating results and fitting coefficient, where the determination coefficients were around 0.97, which demonstrated a good agreement with the results. The Equation 8 can be transformed as the following form,

$$k=a\left(\tau \varphi r{\mathrm{\backslash nolimits}}^{2}\right){\mathrm{\backslash nolimits}}^{b}$$ (9)

where *a* and *b* are fitting parameters. This form aligns with the findings of Nishiyama and Yokoyama [35], who reported that *k* = 8.5(*φr*^2^)^1.3^ provided better description of intrinsic permeability than earlier equations. Thus, combing macro- and micro-scale pore parameters offers a reliable estimate of intrinsic permeability and underscores the significant influence of pore structure, especially at the microscale, on flow behaviors.

#### 3.2.2. Water retention curves.

**Fig 7:**
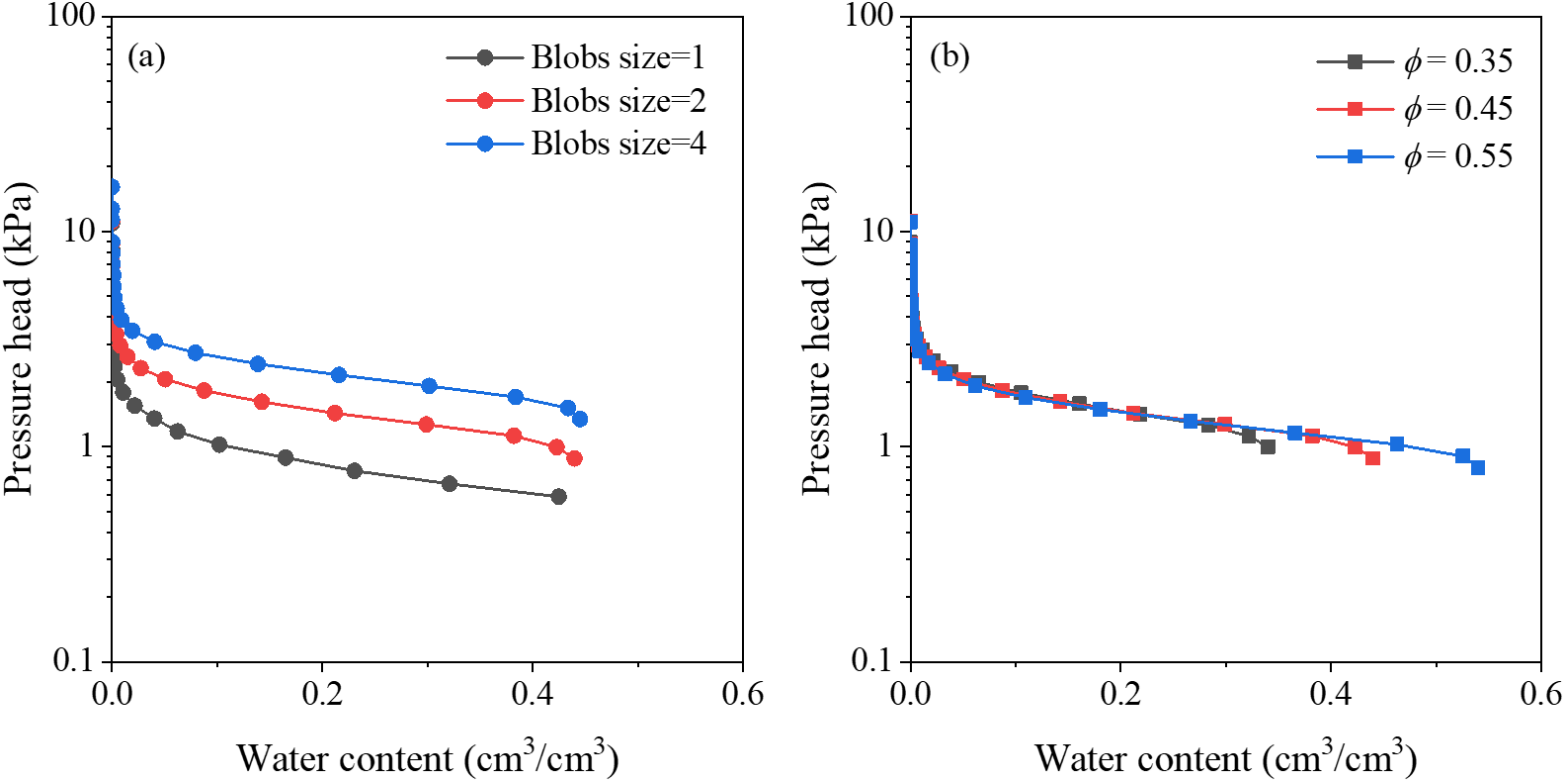
Primary drainage curves for synthetic porous media with various (a) blobs sizes and (b) porosity, respectively.


Water retention curves describe the relationship between water content and pressure head, and reflect pore size distributions. The water-retention curves obtained from pore network simulations for the synthetic porous media are present in Fig 7, and the fitted parameters of the van Genuchten model of Equation 10 are listed in Table 3.

**Table 3:** The fitted van Genuchten parameters of the water retention curve.

Blobs size	Porosity (%)	The van Genuchten parameters		
		*θ*_*s*_ (mm^3^/mm^3^)	*α* (m^-1^)	*n* (-)
1	34.81 ± 0.30	0.39 ± 0.02	12.00 ± 0.69	5.66 ± 0.39
	44.59 ± 0.37	0.48 ± 0.13	14.41 ± 2.12	4.89 ± 0.43
	55.20 ± 0.38	0.56 ± 0.02	15.23 ± 0.27	4.84 ± 0.16
2	34.18 ± 0.13	0.36 ± 0.00	6.38 ± 0.09	6.20 ± 0.58
	44.36 ± 0.24	0.47 ± 0.04	8.19 ± 0.14	4.72 ± 0.44
	54.46 ± 0.25	0.58 ± 0.01	7.88 ± 0.14	5.86 ± 0.03
4	35.14 ± 0.05	0.40 ± 0.03	5.08 ± 0.28	4.63 ± 0.53
	44.68 ± 0.10	0.47 ± 0.05	5.24 ± 0.37	5.54 ± 1.12
	54.17 ± 0.09	0.57 ± 0.00	5.16 ± 0.03	6.67 ± 0.04

$$\theta {\mathrm{\backslash nolimits}}_{w}=\frac{\theta {\mathrm{\backslash nolimits}}_{s}-\theta {\mathrm{\backslash nolimits}}_{r}}{\left[1+\left|\alpha h\right|{\mathrm{\backslash nolimits}}^{n}\right]{\mathrm{\backslash nolimits}}^{1-\frac{\text{1}}{n}}}+\theta {\mathrm{\backslash nolimits}}_{r}$$ (10)

where *θ*_*w*_ is the volumetric water content, *θ*_*s*_ is the saturated water content, *θ*_*r*_ is the residual water content which is approximately equal to zero, and *h* is the pressure head. Traditionally, the parameters *α* is related to the inverse of the air entry suction, and *n* is a measure of pore size distribution [26]. The fitted values of *θ*_*s*_ closely match the porosity of synthetic porous media. The largest values of *α* were occurred in coarse-grained media (12.00–15.23 m^-1^), followed by medium-grained (6.38–8.19 m^-1^), and fine-grained (5.08–5.24 m^-1^) samples. This trend agrees with earlier studies showing that parameter *α* decreases as the proportion of coarse grain (or sand) decreases [36]. Essentially, larger grains produce larger pore radius and throat radius, which strongly influence the entry pressure. As shown in Fig 8, the parameter *α* was in exponential relationship with throat diameter (or pore diameter) in micro scale. In contract, the parameter *n* did not show clear trend across various synthetic media, consistent with several previous studies [37,38]. This may be attributed to the relatively narrow pore size distribution in the generated media: the ratio of largest to smallest pore size here is below 10^3^ (Figs 4a and c), hence, the *n* values are clustered within a narrow interval and show little sensitivity to grain‑size or porosity changes. The insensitivity of *n* in this study serves as a reminder that the van Genuchten model, while widely used, may not always distinguish subtle structural differences when pore-size ranges are limited. In applications where *n* is a critical input, users should verify whether the underlying pore-structure variability justifies the use of a unique *n* value. For materials with similarly constrained pore-size distributions, assuming a constant *n* may be acceptable; however, for natural soils or fractured rocks with pore size ratios often exceeding 10^5^ [15], *n* should be calibrated together with other structural descriptors to avoid misleading predictions.

**Fig 8:**
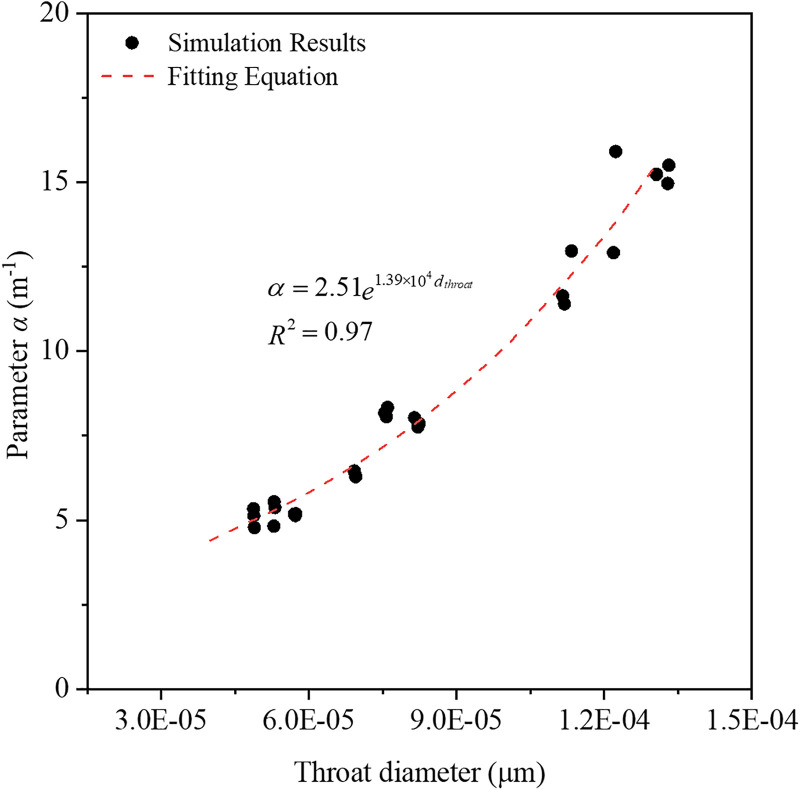
Variation of parameter *α* with the throat diameter.

### 3.3. Two phase of water and gas transport characteristics

#### 3.3.1. Water-gas relative permeability.

**Fig 9:**
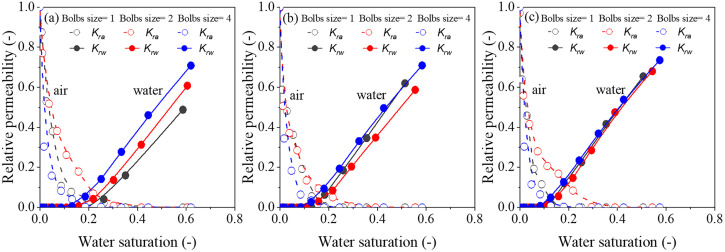
The relative permeability curve for synthetic porous media with porosity of (a) 0.35, (b) 0.45 and (c) 0.55, respectively.

**Fig 10:**
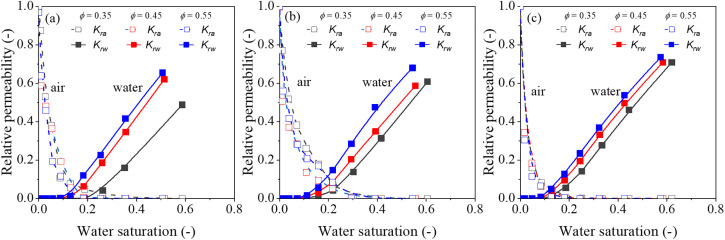
The relative permeability curve for synthetic porous media with blobs sizes of (a) 1, (b) 2 and (c) 4, respectively.

The water-gas relative permeability is an important parameter to evaluate multi-phase flow in porous media, influenced by wettability, pore size distribution, connectivity, and other structural factors. In this stud, the evolution of gas-water relative permeability with varying water saturation was explored through performing pore network modeling. Figs 9 and 10 illustrate the estimated curves of water-gas relative permeability. The relative permeabilities of water and gas are not complementary; that is, *k*_*rw*_ ≠ 1- *k*_*ra*_ due to the gas/water blockage. A sharp decline in gas relative permeability is observed as water saturation increases (typically below 0.2), whereas water relative permeability does not changed obviously, a trend consistent with Mohammadmoradi et al [39] and Wang et al [40]. This may be caused by coordination numbers, that with broader distribution implies greater heterogeneity in local connectivity. Pores with high coordination (well-connected nodes) tend to form preferential pathways for the gas phase, especially at low water saturations. In contrast, pores with low coordination (e.g., coordination ≤ 2) often act as dead-ends or bottlenecks, trapping the water and reducing its effective mobility. This topological heterogeneity explains why the gas relative permeability declines sharply at low water saturations, while the water relative permeability remains nearly unchanged, whereas water remains stranded in poorly-connected pores. In addition, traditional bundle of tubes models or averaged network descriptors often overlook the distribution of coordination numbers, thereby underestimating the trapping capacity and relative permeability hysteresis. The results here highlight that accurate prediction of two-phase flow, especially in media with wide grain-size distributions, requires explicit consideration of the full coordination number distribution rather than its mean value alone. Future pore network models could incorporate coordination-dependent threshold pressures or trapping rules to better capture the observed relative permeability behavior.

The critical water saturation, defined as the saturation below which no significant water flow occurs and only gas flows in porous media [41], varies among the different porous media (Figs 9 and 10). But these differences are relatively small across the samples examined. This limited variation may be attributed to the comparatively large throat diameter in present, where averaged 112.28–132.22 μm for the coarse-grained, 69.35–82 μm for medium-grained, and 48.83–57.22 μm for fine-grained structures (Table 1). Similar behavior was reported by Wang et al [42], who found that a larger frequency percentage of pores in the 10–20 μm range elevated the critical water saturation above 0.3. Because the drastic damage of pore structure would obviously increase gas entry pressure and further hinder water from flowing through the pore space. Consequently, critical water saturation is mainly dominated through the micro pore, which have a decisive role in gas and water entry pressure.

#### 3.3.2. Relative gas diffusivity.

**Fig 11:**
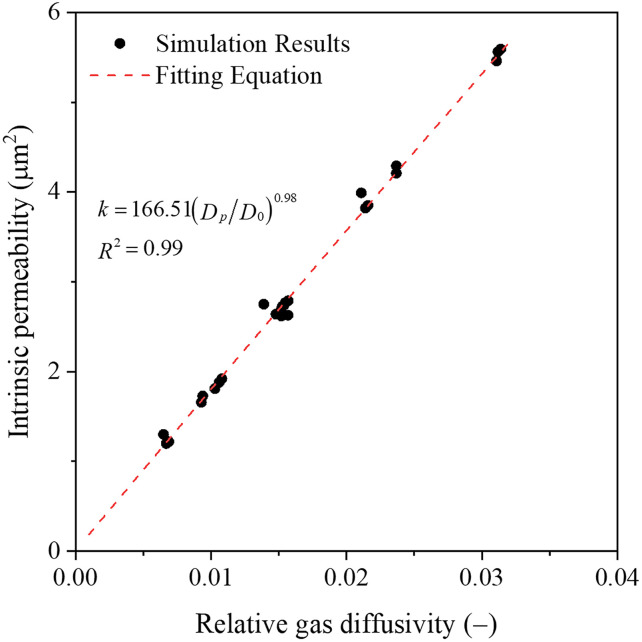
Relationship of relative gas diffusivity and intrinsic permeability.

Gas diffusivity in porous media is the most important parameter describing diffusion-controlled gas migration in porous media, which was estimated by pore network model with effective computation. Table 2 presents the relative gas diffusivity in vertical (*Z*) and horizontal (*X* and *Y*) directions. Like the behavior of intrinsic permeability, the variation of relative gas diffusivity ranged from 0.65 × 10^−2^ to 3.14 × 10^−2^. Thereinto, the largest value (3.11 × 10^−2^ in vertical direction) was observed for coarse‑grained media with the highest porosity (blob size = 1, porosity = 0.55), while the lowest value (0.65 × 10^−2^ in vertical direction) occurred in fine-grained media with the lowest porosity (blobs size = 4 and porosity = 0.35). Relative gas diffusivity also follows a power-law relation with pore-structural parameters for porous media, including porosity and pore (or throat) radius. As shown in Fig 11, the fitted equations exhibit distinct coefficients for different grain sizes, indicating that higher porosity or larger grain sizes provide more continuous pathways for gas diffusion. In essential, the mean free path of gas molecule is typically much smaller than the characteristic pore width, grain-scale attributes such as shape, surface or size have little influence on gas diffusivity. Instead, the dominant controlling factors are pore-structural properties, particularly porosity and pore connectivity [43,44].

Intrinsic permeability and relative gas diffusivity exhibit similar trends with respect to porosity and throat radius. Thus, various expressions related to intrinsic permeability and relative gas diffusivity have been found in the literatures [6,45], most of which follow in a power regression model, i.e., *k* = *m*(*D*_*p*_/*D*_*0*_)^*w*^, and constats *m* and *w* vary with porous materials. For the synthetic media here, the correlation can be expressed as,


$$k=166.51\left(D{\mathrm{\backslash nolimits}}_{p}/D{\mathrm{\backslash nolimits}}_{0}\right){\mathrm{\backslash nolimits}}^{0.98}$$ (11)

where the squared correlation coefficient of regression is 0.99. Using intrinsic permeability to estimate gas diffusivity can greatly simplify the characterization of gas transport in porous materials. Although Equation 11 provides a concise and well fitted correlation between intrinsic permeability and relative gas diffusivity for the synthetic media studied here, several important limitations must be acknowledged when considering its practical application. In actual, the constants of *m* and *w* closely depend on porous media types and external environment; therefore, predicting relative gas diffusivity solely from permeability is not universally accurate. The synthetic media were generated using a well-controlled “blobs” model with relatively narrow pore size distributions and simplified pore shapes (spheres and cylinders) in present study. Natural porous systems (e.g., soils, sedimentary rocks, bio scaffolds) often exhibit wider pore size ranges, more complex pore morphologies, and heterogeneous connectivity, which can significantly alter the exponent *w* and pre factor *m*. For example, studies on soils and cementitious materials report exponents *w* ranged from 1.3 to 2.5 [45,46]. In addition, in field applications, factors such as temperature gradients, multiphase coexistence, and reactive transport can modify the effective diffusion path and thus decouple the permeability-diffusivity correlation. Hence, the suggested expression of Equation 11 should be applied only to porous media with structural characteristics like those examined in this work.

## 4. Conclusions

This study investigates f the pore structure characteristic of synthetic porous media and their effect on flow and transport properties. Synthetic porous media with varying grain sizes (blob sizes) and porosity levels were generated, and their pore structures were extracted from 3D images and characterized by pore networks. The effect of pore structure on key flow characteristics of porous media, including intrinsic permeability, water retention curve, water-gas relative permeability and relative gas diffusivity, were quantitatively evaluated through pore network model.

The extracted pore networks clearly captured the influence of grain size on pore-structure features. Tortuosity decreased with increasing porosity but increased with grain size. For a given porosity, coarse-grained media exhibited larger mean pore diameter, throat diameter, and throat length compared to fine-grained one. In contrast, pore number, throat number, and coordination number decreased obviously as grain size increased. The distribution of pore diameter, throat diameter, throat length and coordination number all followed lognormal distribution. Intrinsic permeability was higher in coarse‑grained media and increased with porosity. A power-law relationship combining the macroscale parameter porosity and the microscale parameters pore (or throat) radius provided a reliable estimation of intrinsic permeability. The water-retention curves obtained from pore network simulations showed that the van Genuchten parameter *α* exhibited an exponential dependence on throat (or pore) diameter in microscale, while parameter *n* displayed no clear trend across different media due to the relatively narrow pore size distribution. Similarly, for water-gas relative permeability, the critical water saturation varied only slightly among different porous media. Relative gas diffusivity also followed a power-law relationship with porosity and pore/throat radius. Furthermore, a strong correlation between intrinsic permeability and relative gas diffusivity was established as *k* = 166.51(*D*_*p*_/*D*_*0*_)^0.98^, offering a direct means to estimate relative gas diffusion from intrinsic permeability measurements.

## Supporting information

S1 Data Supporting data for this study. (XLSX)

## References

[1] Chen L, He A, Zhao J, Kang Q, Li Z-Y, Carmeliet J, et al. Pore-scale modeling of complex transport phenomena in porous media. Prog Energy Combust Sci. 2022;88:100968. doi: 10.1016/j.pecs.2021.100968

[2] Meigel FJ, Darwent T, Bastin L, Goehring L, Alim K. Dispersive transport dynamics in porous media emerge from local correlations. Nat Commun. 2022;13(1):5885. doi: 10.1038/s41467-022-33485-5 36202817 PMC9537155

[3] Hamamoto S, Ohko Y, Ohtake Y, Moldrup P, Nishimura T. Water‐ and air‐filled pore networks and transport parameters under drying and wetting processes. Vadose Zone J. 2022;21(4). doi: 10.1002/vzj2.20205

[4] Dai J, Zhang G, Liu L, Shi P, Zhang H, Han X, et al. Effects of efflorescence and subflorescence by different salts on soil physical properties and aeolian erosion. CATENA. 2022;215:106323. doi: 10.1016/j.catena.2022.106323

[5] Caplan JS, Giménez D, Subroy V, Heck RJ, Prior SA, Runion GB, et al. Nitrogen-mediated effects of elevated CO2 on intra-aggregate soil pore structure. Glob Chang Biol. 2017;23(4):1585–97. doi: 10.1111/gcb.13496 27726258

[6] Katuwal S, Arthur E, Tuller M, Moldrup P, de Jonge LW. Quantification of soil pore network complexity with X-ray computed tomography and gas transport measurements. Soil Sci Soc Am J. 2015;79(6):1577–89. doi: 10.2136/sssaj2015.06.0227

[7] Chang C, Cheng D, Qiao X. Improving estimation of pore size distribution to predict the soil water retention curve from its particle size distribution. Geoderma. 2019;340:206–12. doi: 10.1016/j.geoderma.2019.01.011

[8] Cai Y, Li Q, Liu D, Zhou Y, Lv D. Insights into matrix compressibility of coals by mercury intrusion porosimetry and N2 adsorption. Int J Coal Geol. 2018;200:199–212. doi: 10.1016/j.coal.2018.11.007

[9] Qin L, Li S, Zhai C, Lin H, Zhao P, Yan M, et al. Joint analysis of pores in low, intermediate, and high rank coals using mercury intrusion, nitrogen adsorption, and nuclear magnetic resonance. Powder Technol. 2020;362:615–27. doi: 10.1016/j.powtec.2019.12.019

[10] Xiong Q, Baychev TG, Jivkov AP. Review of pore network modelling of porous media: Experimental characterisations, network constructions and applications to reactive transport. J Contam Hydrol. 2016;192:101–17. doi: 10.1016/j.jconhyd.2016.07.002 27442725

[11] Qie Z, Rabbani A, Liang Y, Sun F, Behnsen J, Wang Y, et al. Multiscale investigation of pore network heterogeneity and permeability of fluid catalytic cracking (FCC) particles. Chem Eng J. 2022;440:135843. doi: 10.1016/j.cej.2022.135843

[12] Qin X, Xia Y, Wu J, Sun C, Zeng J, Xu K, et al. Influence of Pore Morphology on Permeability through Digital Rock Modeling: New Insights from the Euler Number and Shape Factor. Energy Fuels. 2022;36(14):7519–30. doi: 10.1021/acs.energyfuels.2c01359

[13] Daneshian B, Habibagahi G, Nikooee E. Determination of unsaturated hydraulic conductivity of sandy soils: A new pore network approach. Acta Geotech. 2020;16(2):449–66. doi: 10.1007/s11440-020-01088-3

[14] Li X, Teng Q, Zhang Y, Xiong S, Feng J. Three-dimensional multiscale fusion for porous media on microtomography images of different resolutions. Phys Rev E. 2020;101(5–1):053308. doi: 10.1103/PhysRevE.101.053308 32575196

[15] Mufti S, Das A. Multiscale pore network construction for two phase flow simulations in granular soils. Adv Water Resour. 2023;173:104386. doi: 10.1016/j.advwatres.2023.104386

[16] Metzger T. A personal view on pore network models in drying technology. Drying Technol. 2018;37(5):497–512. doi: 10.1080/07373937.2018.1512502

[17] Turturro AC, Caputo MC, Gerke HH. Mercury intrusion porosimetry and centrifuge methods for extended‐range retention curves of soil and porous rock samples. Vadose Zone J. 2021;21(1). doi: 10.1002/vzj2.20176

[18] Luo L, Lin H, Li S. Quantification of 3-D soil macropore networks in different soil types and land uses using computed tomography. J Hydrology. 2010;393(1–2):53–64. doi: 10.1016/j.jhydrol.2010.03.031

[19] Ghanbarian B, Hunt AG, Skaggs TH, Jarvis N. Upscaling soil saturated hydraulic conductivity from pore throat characteristics. Adv Water Resour. 2017;104:105–13. doi: 10.1016/j.advwatres.2017.03.016

[20] Li G, Zhan L, Yun T, Dai S. Pore‐scale controls on the gas and water transport in hydrate‐bearing sediments. Geophys Res Lett. 2020;47(12). doi: 10.1029/2020gl086990

[21] Wei Y, Chen K, Wu J. Estimation of the critical infiltration rate for air compression during infiltration. Water Resour Res. 2020;56(3). doi: 10.1029/2019wr026410

[22] Gostick JT. Versatile and efficient pore network extraction method using marker-based watershed segmentation. Phys Rev E. 2017;96(2–1):023307. doi: 10.1103/PhysRevE.96.023307 28950550

[23] Gostick J, Khan Z, Tranter T, Kok M, Agnaou M, Sadeghi M, et al. PoreSpy: A Python Toolkit for quantitative analysis of porous media images. JOSS. 2019;4(37):1296. doi: 10.21105/joss.01296

[24] Gostick J, Aghighi M, Hinebaugh J, Tranter T, Hoeh MA, Day H, et al. OpenPNM: A pore network modeling package. Comput Sci Eng. 2016;18(4):60–74.

[25] Zhang B, Zhao B, Sun Z. Pore-scale investigation of drying dynamics and salt precipitation forms in porous media. Adv Water Resour. 2025;206:105147. doi: 10.1016/j.advwatres.2025.105147

[26] van Genuchten MTh. A closed‐form equation for predicting the hydraulic conductivity of unsaturated soils. Soil Sci Soc of Am J. 1980;44(5):892–8. doi: 10.2136/sssaj1980.03615995004400050002x

[27] Hamamoto S, Moldrup P, Kawamoto K, Sakaki T, Nishimura T, Komatsu T. Pore network structure linked by X-ray CT to particle characteristics and transport parameters. Soils Foundations. 2016;56(4):676–90. doi: 10.1016/j.sandf.2016.07.008

[28] Xiong Y, Dong L, Long X, Chen M, Huang G. Pore-network model to quantify internal structure and hydraulic characteristics of randomly packed grains with different morphologies. Granular Matter. 2021;24(1). doi: 10.1007/s10035-021-01174-7

[29] Ren XW, Santamarina JC. The hydraulic conductivity of sediments: A pore size perspective. Eng Geol. 2018;233:48–54. doi: 10.1016/j.enggeo.2017.11.022

[30] Dong L, Zhang W, Xiong Y, Zou J, Huang Q, Xu X, et al. Impact of short-term organic amendments incorporation on soil structure and hydrology in semiarid agricultural lands. Int Soil Water Conservation Res. 2022;10(3):457–69. doi: 10.1016/j.iswcr.2021.10.003

[31] Liu B, Ma R, Fan H. Evaluation of the impact of freeze-thaw cycles on pore structure characteristics of black soil using X-ray computed tomography. Soil Tillage Res. 2021;206:104810. doi: 10.1016/j.still.2020.104810

[32] Huang X, He Y, Zhou W, Deng D, Zhao Y. Pore network modeling of fibrous porous media of uniform and gradient porosity. Powder Technol. 2019;343:350–61. doi: 10.1016/j.powtec.2018.11.022

[33] Liu YF, Jeng D-S. Pore scale study of the influence of particle geometry on soil permeability. Adv Water Resour. 2019;129:232–49. doi: 10.1016/j.advwatres.2019.05.024

[34] Gharedaghloo B, Price JS, Rezanezhad F, Quinton WL. Evaluating the hydraulic and transport properties of peat soil using pore network modeling and X-ray micro computed tomography. J Hydrology. 2018;561:494–508. doi: 10.1016/j.jhydrol.2018.04.007

[35] Nishiyama N, Yokoyama T. Permeability of porous media: Role of the critical pore size. JGR Solid Earth. 2017;122(9):6955–71. doi: 10.1002/2016jb013793

[36] Tian Z, Gao W, Kool D, Ren T, Horton R, Heitman JL. Approaches for estimating soil water retention curves at various bulk densities with the extended van genuchten model. Water Resour Res. 2018;54(8):5584–601. doi: 10.1029/2018wr022871

[37] Richard G, Sillon JF, Marloie O. Comparison of inverse and direct evaporation methods for estimating soil hydraulic properties under different tillage practices. Soil Sci Soc Am J. 2001;65(1):215–24. doi: 10.2136/sssaj2001.651215x

[38] Stange CF, Horn R. Modeling the soil water retention curve for conditions of variable porosity. Vadose Zone J. 2005;4(3):602–13. doi: 10.2136/vzj2004.0150

[39] Mohammadmoradi P, Kantzas A. Direct geometrical simulation of pore space evolution through hydrate dissociation in methane hydrate reservoirs. Marine Petroleum Geology. 2018;89:786–98. doi: 10.1016/j.marpetgeo.2017.11.016

[40] Wang J, Zhao J, Zhang Y, Wang D, Li Y, Song Y. Analysis of the effect of particle size on permeability in hydrate-bearing porous media using pore network models combined with CT. Fuel. 2016;163:34–40. doi: 10.1016/j.fuel.2015.09.044

[41] Badawy AM, Ganat TAAO. Fluid Saturation. Rock Properties and Reservoir Engineering: A Practical View. Cham: Springer International Publishing; 2022. 29–34.

[42] Wang D, Wang C, Li C, Liu C, Lu H, Wu N, et al. Effect of gas hydrate formation and decomposition on flow properties of fine-grained quartz sand sediments using X-ray CT based pore network model simulation. Fuel. 2018;226:516–26. doi: 10.1016/j.fuel.2018.04.042

[43] Fu Z, Yan Z, Li S. Effects of soil pore structure on gas diffusivity under different land uses: Characterization and modelling. Soil Tillage Res. 2024;237:105988. doi: 10.1016/j.still.2023.105988

[44] Talukder R, Plaza-Bonilla D, Cantero-Martínez C, Wendroth O, Castel JL. Soil gas diffusivity and pore continuity dynamics under different tillage and crop sequences in an irrigated Mediterranean area. Soil Tillage Res. 2022;221:105409. doi: 10.1016/j.still.2022.105409

[45] Benavente D, Pla C. Effect of pore structure and moisture content on gas diffusion and permeability in porous building stones. Mater Struct. 2018;51(1). doi: 10.1617/s11527-018-1153-8

[46] Klink T, Gaber K, Schlattner E, Setzer MJ. Characterisation of the gas transport properties of porous materials by determining the radon diffusion coefficient. Mater Struct. 1999;32(10):749–54. doi: 10.1007/bf02905071

